# Obstructive sleep apnea in patients surviving acute hypercapnic respiratory failure is best predicted by static hyperinflation

**DOI:** 10.1371/journal.pone.0205669

**Published:** 2018-10-25

**Authors:** Dan Adler, Elise Dupuis-Lozeron, Jean Paul Janssens, Paola M. Soccal, Frédéric Lador, Laurent Brochard, Jean-Louis Pépin

**Affiliations:** 1 Service de Pneumologie, Département des spécialités de médecine, Geneva University Hospitals, Geneva, Switzerland; 2 University of Geneva Faculty of Medicine, Geneva, Switzerland; 3 Division d’épidémiologie clinique, Geneva University Hospitals, Geneva, Switzerland; 4 Keenan Research Center and Li Ka Shing Knowledge Institute, Department of Critical Care, St Michael’s Hospital, Toronto, Canada; 5 Interdepartmental Division of Critical Care Medicine, University of Toronto, Toronto, Canada; 6 Laboratoire HP2, Inserm 1042, Université Grenoble Alpes, Grenoble, France; National Taiwan University Hospital, TAIWAN

## Abstract

**Rationale:**

Acute hypercapnic respiratory failure (AHRF) treated with non-invasive ventilation in the ICU is frequently caused by chronic obstructive pulmonary disease (COPD) exacerbations and obesity-hypoventilation syndrome, the latter being most often associated with obstructive sleep apnea. Overlap syndrome (a combination of COPD and obstructive sleep apnea) may represent a major burden in this population, and specific diagnostic pathways are needed to improve its detection early after ICU discharge.

**Objectives:**

To evaluate whether pulmonary function tests can identify a high probability of obstructive sleep apnea in AHRF survivors and outperform common screening questionnaires to identify the disorder.

**Methods:**

Fifty-three patients surviving AHRF (31 males; median age 67 years (interquartile range: 62–74) participated in the study. Anthropometric data were recorded and body plethysmography was performed 15 days after ICU discharge. A sleep study was performed 3 months after ICU discharge.

**Results:**

The apnea-hypopnea index was negatively associated with static hyperinflation as measured by the residual volume to total lung capacity ratio in the % of predicted (coefficient = -0.64; standard error 0.17; 95% CI -0.97 to -0.31; p<0.001). A similar association was observed in COPD patients only: coefficient = -0.65; standard error 0.19; 95% CI -1.03 to -0.26; p = 0.002. Multivariate analysis with penalized maximum likelihood confirmed that the residual volume to total lung capacity ratio was the main contributor for apnea-hypopnea index variance in addition to classic predictors. Screening questionnaires to select patients at risk for sleep-disordered breathing did not perform well.

**Conclusions:**

In AHRF survivors, static hyperinflation is negatively associated with the apnea-hypopnea index in both COPD and non-COPD patients. Measuring static hyperinflation in addition to classic predictors may help to increase the recognition of obstructive sleep apnea as common screening tools are of limited value in this specific population.

## Introduction

Chronic obstructive pulmonary disease (COPD) and obstructive sleep apnea (OSA) are highly prevalent chronic diseases and represent a considerable economic burden for health care systems worldwide [1–3]. Acute hypercapnic respiratory failure (AHRF) in patients admitted to the intensive care unit (ICU) is mainly due to COPD exacerbation or obesity-hypoventilation syndrome. In a high proportion of these individuals, non-invasive ventilation treatment is successful [4–7]. OSA is a component of the obesity-hypoventilation syndrome in more than 85% of cases and most patients with a clinical diagnosis of this syndrome remain treated in the long term with non-invasive positive airway pressure [5, 8, 9]. Conversely, only selected subgroups of COPD patients with persistent hypercapnia after AHRF benefit from home non-invasive ventilation as evidenced by recent high-quality randomized controlled trials [10, 11]. Cohort studies also suggest that untreated COPD patients with co-morbid OSA are at increased risk for repeated exacerbations and death [12, 13].

The combination of COPD and OSA (the so-called “overlap syndrome”) is characterized by more pronounced nocturnal hypoxemia and a higher risk of pulmonary hypertension and right heart failure [14–17]. Overlap syndrome prevalence is low in the general population [18, 19], but it represents a major burden in AHRF survivors [7]. The combination of a cluster of comorbidities, including OSA and COPD, has been associated with a higher risk of death and early readmission after discharge for AHRF [7]. This implies that specific diagnostic pathways targeting overlap syndrome should be implemented in this specific AHRF population for its early identification and treatment in order to improve long-term outcomes.

While a suspicion of OSA in an obese population is obvious, this may not be the case in AHRF patients. In addition, COPD is largely underdiagnosed in this population and pulmonary function tests are recommended following AHRF [7]. The primary objective of this study was to evaluate whether a systematic assessment of pulmonary function tests, including a measure of static hyperinflation, would increase the pretest probability of having OSA in patients surviving AHRF, thus allowing to improve patient selection for polysomnography. Our secondary objective was to assess whether pulmonary function tests would outperform common screening questionnaires used to identify at-risk patients in this specific population.

## Methods

### Study population

We performed a single-center, prospective, cohort study of patients surviving AHRF at the Geneva University Hospitals (Geneva, Switzerland) from 2012–2015. Precise inclusion and exclusion criteria have been described previously, as well as a detailed description of objectively assessed major comorbidities associated with AHRF [7]. In brief, 197 patients who had survived AHRF were screened for inclusion in the study at ICU discharge. Exclusion criteria were: unwillingness to participate in a clinical study (39%); confusion or psychiatric comorbidities (22%); life expectancy < 3 months (18%); premature transfer to another facility (12%); and not meeting inclusion criteria (9%). Among 78 patients included, only those (n = 53) who completed an overnight sleep study after ICU discharge were included in the analysis. All patients provided written informed consent and the study was approved by the institutional review board (Comité départemental d’éthique des spécialités médicales et de médecine communautaire et de premier recours, Geneva University Hospitals, Geneva, Switzerland, CER11-28) and registered at http://www.clinicaltrials.gov (identifier NCT02111876).

### Sleep studies

Polysomnography or cardiorespiratory recordings were performed 3 months after ICU discharge while the patient was spontaneously breathing 2 days after withdrawal of positive airway pressure treatment. Sleep studies were performed during a phase of clinical stability assessed by a study nurse using a standardized interview followed by a physical examination. Sleep stages and arousals were scored using the American Academy of Sleep Medicine criteria. Apnea was scored if a drop of 90% or more in airflow signal excursion was noted for at least 10 sec. Hypopnea was defined as a drop greater than or equal to 30% in airflow lasting at least 10 sec and associated with 3% oxygen desaturation [20]. An apnea-hypopnea index (AHI) cut-off value >20/h was used for moderate-to -severe sleep apnea as this cut-off has recently been associated with both hypertension and metabolic syndrome after multiple adjustment for risk factors [21, 22].

### Pulmonary function tests

Standardized measurements of lung volumes using body plethysmography was performed 15 days after ICU discharge in all patients. A forced expiratory volume in 1 sec (FEV1) over the forced vital capacity ratio (FVC) of less than the 5^th^ percentile of the predicted value was used to define obstructive lung disease. Residual volume (RV) to total lung capacity ratio (TLC) (% of predicted) was used to assess the degree of static hyperinflation as proposed by the American Thoracic Society/European Respiratory Society 2005 taskforce [23]. All measures were performed after administration of short-acting beta-agonist bronchodilators.

### Questionnaire and score for OSA

STOP-Bang, an 8-item questionnaire combining symptoms and clinical variables, was obtained for all patients 3 months after ICU discharge as a screening tool for OSA identification [24]. A threshold of 5 points or more was used to identify patients at risk for moderate-to-severe sleep-disordered breathing. The NoSAS score was computed for all patients using the variables available in our database (neck circumference, body mass index (BMI), snoring, age, gender) [22]. A threshold of 8 points or more was used to identify patients at risk for moderate-to-severe sleep-disordered breathing.

### Statistical analysis

Descriptive statistics are reported as numbers and percentages for categorical variables or the median and interquartile range (IQR) for continuous variables. When comparing groups, we used the Mann-Whitney-Wilcoxon test for continuous variables or Fishers’ exact test for categorical variables. Univariate analysis to predict the AHI was conducted by using a linear regression model. Ridge regression [25] (a shrinkage method that adjusts the model parameters for overoptimism) was used to develop predictive models of AHI. The penalty factor was chosen by maximizing the modified Akaike’s information criteria [26]. The full penalized model was then simplified using the method proposed by Ambler and Moons [27, 28]. AHI predicted by the simplified model was then used to estimate moderate-to-severe sleep apnea. Sensitivity, specificity, and negative and positive predictive values, as well as the area under the curve, were computed. All analyses were performed using R version 3.4.2 (R Project for Statistical Computing, Vienna, Austria) and the rms package [29]. P values less than 0.05 were considered significant.

## Results

Baseline characteristics of the study population are shown in Table 1. Fifty-three patients surviving AHRF were enrolled in the study (31 males; median age 67 years [IQR 62–74]; BMI 33 [26–41] kg/m^2^). The prevalence of COPD was high with no difference between patients with or without significant OSA (64% versus 82% respectively; p = 0.296).

**Table 1:** Patient characteristics.

	Overall (n = 53)	Moderate-to-severe OSA (n = 36)	No or mild OSA (n = 17)	p-value
Gender, female/male	22/31	15/21	7/10	1.000
Age (yrs)	67 (62–74)	66 (62–72)	71 (64–75)	0.311
COPD, n (%)	37 (70)	23 (64)	14 (82)	0.296
FEV1, % of predicted value	50 (39–58)	54 (44–64)	39 (27–45)	0.001
FEV1/FVC, %	57 (46–73)	65 (51–76)	51 (39–67)	0.073
TLC, % of predicted value	84 (67–104)	85 (65–98)	83 (68–113)	0.641
RV, % of predicted value	111 (81–143)	99 (81–140)	120 (101–176)	0.092
RV/TLC, % of predicted value	130 (114–150)	121 (112–138)	149 (139–162)	0.002
AHI,n /h	36 (17–58)	48 (36–76)	9 (5–16)	<0.001
BMI, kg/m2	33 (26–41)	33 (29–41)	27 (23–39)	0.042
Neck circumference, cm	43 (38–46)	43 (40–47)	41 (37–46)	0.211
Hypertension, n (%)	33/53(62)	22/36 (61)	11/17 (65)	1.00
Epworth Sleepiness Scale	7 (4–12)	7 (4–12)	7 (5–9)	0.709

OSA: obstructive sleep apnea; COPD: chronic obstructive lung disease; FEV1: forced expiratory volume in 1 sec.; FVC: forced vital capacity; RV: residual volume; TLC: total lung capacity; AHI; apnea-hyponea index; BMI: body mass index

### Association between hyperinflation and the severity of sleep-disordered breathing

**Fig 1:**
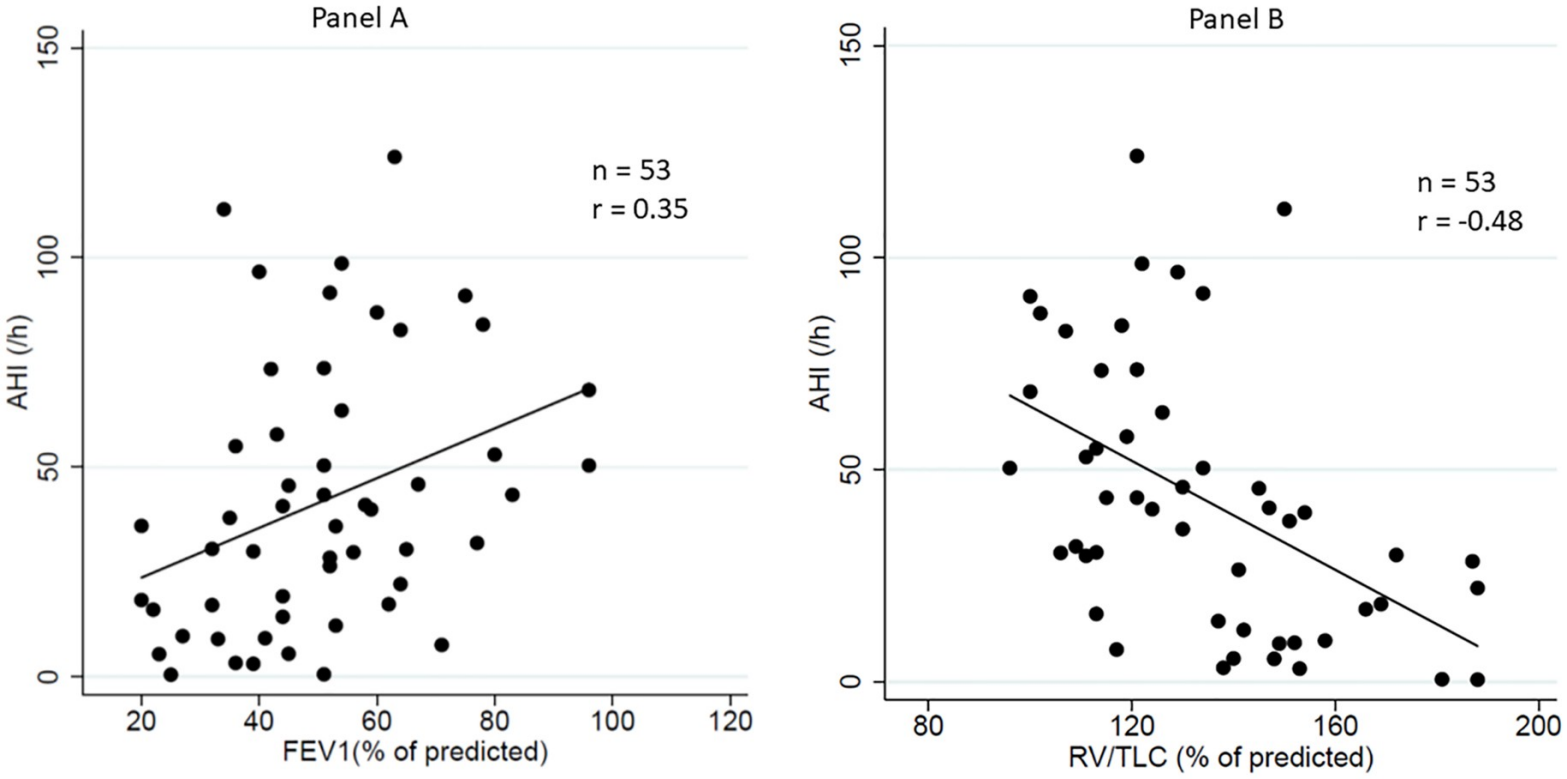
Panel A: Positive correlation between FEV1 (% of predicted) and the apnea-hypopnea index. Panel B: Negative correlation between residual volume/total lung capacity (% of predicted) and the apnea-hypopnea index. FEV1: forced expiratory volume in 1 sec;.

Overall, the AHI was negatively associated with static hyperinflation as measured by the % of the predicted RV/TLC ratio (coefficient = - 0.64; SE 0.17; 95% CI -0.97 to -0.31; p<0.001) and positively associated with the % of the predicted value of FEV1 (coefficient = 0.59; SE 0.23; 95% CI 0.14 to 1.05; p = 0.012 [data previously reported [7]). Similar associations between lung volume and the AHI were observed in the specific COPD subgroup (coefficient = -0.65; SE 0.19; 95% CI -1.03 to -0.26; p = 0.002 for the % of the predicted RV/TLC ratio; and coefficient = 0.75; SE 0.31; 95% CI 0.09 to 1.37; p = 0.026 for FEV1). (Fig 1).

### Predictors of sleep-disordered breathing

In univariate analysis, there was a positive association between AHI and body mass index (coefficient = 1.04; SE 0.41; 95% CI 0.21 to 1.87; p = 0.015), neck circumference (coefficient = 2.07; SE 0.71; 95% CI 0.64 to 3.50; p = 0.005), and the Epworth Sleep Scale(coefficient = 2.12; SE 0.94; 95% CI 0.24 to 4.00; p = 0.028), but not with age (coefficient = -0.54; SE 0.45; 95% CI -1.44 to -0.37; p = 0.238), gender (coefficient = 14.23; SE 8.64; 95% CI -3.12 to 31.58; p = 0.106) and hypertension (coefficient = 3.22; SE 9.01; 95% CI -14.86 to 21.30; p = 0.722).

**Fig 2:**
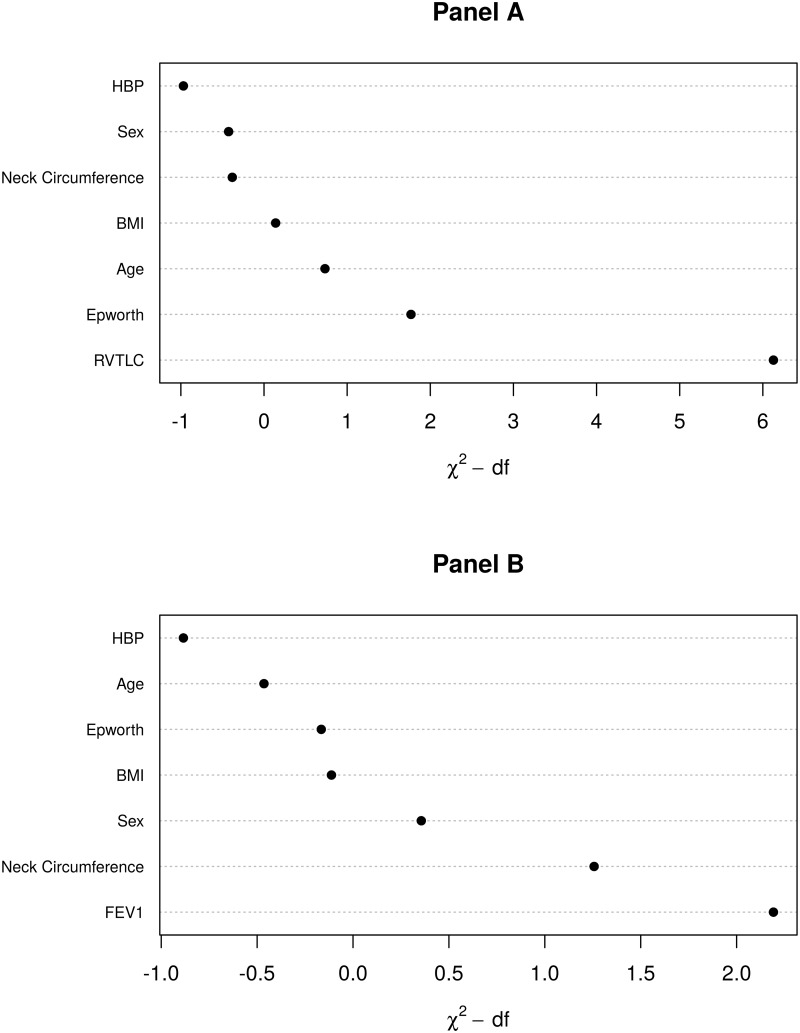
Panels A and B: Relative importance of each predictor in the full penalized model measured by the partial Wald chi-square minus degrees of freedom. Higher values indicate more important variables.

In multivariate analysis, the penalized maximum likelihood revealed that RV/TLC (% of predicted) or FEV1 (% of predicted) was the main contributor for AHI variance, in addition to classic predictors (Table 2 and Fig 2). In this particular population, screening questionnaires to select patients at risk for sleep-disordered breathing did not perform well (Table 3).

**Table 2:** Multivariate analysis of the apnea-hypopnea index.

a. Multivariate analysis of the apnea-hypopnea index with residual value/total lung capacity (% of predicted) as an independent variable.					
Apnea-hypopnea index (/h)	Regression coefficient	Standard error	p-value	95% confidence	interval
Neck circumference (cm)	1.38	0.60	0.352	-0.65	1.78
RV/TLC (% of predicted)	-0.37	0.14	0.010	-0.65	-0.09
Epworth Sleepiness Scale	1.38	0.74	0.069	-0.11	2.86
Age	-0.46	0.32	0.161	-1.11	0.19
BMI	0.33	0.37	0.366	-0.40	1.07
b. Multivariate analysis of apnea-hypopnea index with FEV1 (% of predicted) as an independent variable.					
Apnea-hypopnea index (/h)	Regression coefficient	Standard error	p-value	95% confidence	interval
Male gender	5.23	5.24	0.32	-5.30	15.76
Neck circumference (cm)	1.04	0.53	0.057	-0.03	2.11
FEV1 (% of predicted)	0.35	0.17	0.045	0.01	0.68
Epworth Sleepiness Scale	0.72	0.69	0.301	-0.66	2.11
Age	-0.21	0.31	0.504	-0.84	0.42

RV: residual volume; TLC: total lung capacity; BMI: body mass index; FEV1: forced expiratory volume in 1 sec.

**Table 3:** Performance of classic screening tools for obstructive sleep apnea and our 3 variables model.

	Sensitivity (95% CI)	Specificity (95% CI)	Positive predictive value (95% CI)	Negative predictive value (95% CI)	Area under the curve (95% CI)
Simplified penalized regression model with RV/TLC	0.97 (0.85–1)	0.20 (0.04–0.48)	0.74 (0.59–0.86)	0.75 (0.19–0.99)	0.78 (0.65–0.91)
Simplified penalized regression model with FEV1	0.97 (0.85–1)	0.20 (0.04–0.48)	0.74 (0.59–0.86)	0.75 (0.19–0.99)	0.75 (0.60–0.89)
NoSAS	0.89 (0.72–0.96)	0.24 (0.08–0.50)	0.71 (0.55–0.83)	0.50 (0.18–0.83)	0.65 (0.50–0.81)
STOP BANG	0.78 (0.60–0.89)	0.41 (0.19–0.67)	0.74 (0.57–0.86)	0.47 (0.22–0.73)	0.58 (0.42–0.75)

RV: residual volume; TLC: total lung capacity; FEV1: forced expiratory volume in 1 sec.

## Discussion

Our study expands current knowledge of interactions between lung mechanics and sleep-disordered breathing and shows that the severity of static hyperinflation is negatively associated with the AHI in both COPD and non-COPD patients surviving AHRF. Classic screening tools for OSA did not perform well and are of limited interest in this specific population.

Whether the association between COPD and OSA occurs by chance or can be explained by specific mechanisms remains a subject of debate. Upper airway inflammation consecutive to smoking, rostral fluid shift, upper airway muscle weakness and inhaled corticosteroids may promote OSA in COPD patients, while other factors may be protective, such as a low BMI and a diminished rapid-eye movement sleep stage [14]. Apart from the classic risk factors for OSA, lung volume and respiratory mechanics also have a significant impact on upper airway size and patency [30–32]. Hyperinflation and increased lung volume help to stabilize the upper airway as demonstrated by physiological studies in healthy humans [31, 33], as well in OSA [34] and COPD [35]patients. In the latter study, COPD patients exhibited lower passive pharyngeal critical closing pressure than their matched controls, thus meaning a less collapsible upper airway [35]. In another study, a high percentage of air trapping and emphysema assessed by a computed tomography scan was associated with the lowest AHIs [36]. Our data demonstrate that static hyperinflation measured by RV/TLC is negatively associated with AHI in a population (mainly COPD and obese patients) surviving AHRF. This also applies to FEV1, which is easier to measure in routine clinical practice. As already highlighted by others [37], classic predictors of OSA are less or not valid in highly-specific populations, such as AHRF survivors.

OSA is a major global health concern and a cause of considerable cardiovascular and metabolic morbidity and mortality [3]. However, the disorder is a complex, heterogeneous and multi-component condition. In a large cluster analysis of more than 18,000 OSA patients from a prospective national registry, Bailly et al. identified six distinct phenotypes at the time of diagnosis with patients varying considerably in age, BMI, symptoms, comorbidities, and risk exposure [38]. Similarly, all previously published cluster analyses have also emphasized the significant heterogeneity between OSA patients at the time of diagnosis [39, 40]. Accordingly, it can be presumed that predictors of OSA might differ for each phenotype or referral context and, therefore, it is probably simplistic to believe that a “one size fits all” screening questionnaire will be suitable for all patients.

Indeed, in our study population, the Epworth Sleepiness Score was in the normal range for the entire cohort with similar median values for both OSA and non-OSA patients. Likewise, age distribution was too narrow to show differences between groups and the female:male ratio was also similar. Our understanding of this phenomenon is that classic predictors of OSA obtained from an unselected sample of the general population will not perform well in highly-selected homogenous groups of severely ill patients. One strategy to overcome this issue is to increase the use of ambulatory monitoring tools for a simplified OSA diagnosis [41], especially if pre-test probability of OSA is high [7, 42]. A complementary approach is to develop specific screening tools appropriate to the clinical context. For instance, in a population of moderate-to-severe COPD referred for a pulmonary rehabilitation program, a simple COPD-OSA screening test including BMI and a history of cardiovascular disease has recently been demonstrated to be superior to gender, the Epworth Sleepiness Score, and other classic predictors of OSA [37].

Following AHRF, we demonstrated that comorbidities are under-recognized and associated with poor outcome, despite patients being frequently hospitalized^15^. It is therefore timely to validate specific pathways for the assessment of important comorbidities in survivors. Indeed, only a minority of COPD patients presenting with AHRF had previously been diagnosed by spirometry. Based on our data, systematic pulmonary function tests should be recommended for all AHRF survivors as a first step for COPD phenotyping and to give the clinician a hint towards a comorbid diagnosis of OSA.

Our study has some limitations. Although we have demonstrated that pulmonary function tests were important predictors of AHI in AHRF survivors and should probably be included in screening tools, the current sample size in our study population restricted our ability to model predictive equations robust enough to anticipate individual AHIs. In addition, the need to investigate patients by pulmonary function tests might be considered as a limitation compared to classic screening questionnaires. However, complete pulmonary functions tests will also help the clinician to better assess multi-morbidity including OSA. We also acknowledge that our data were obtained from a single-center, prospective cohort of patients surviving ICU stay. Therefore, our results cannot be extended to patients with AHRF who died in the ICU or were lost to follow-up. However, from a clinical point of view, the assessment of multimorbidity can only be performed in the clinic in stable patients who survived the episode of AHRF. Moreover, our study focused on AHRF survivors, so our results cannot be extended to the general population. Although we used a modern statistical method to correct for optimism (shrinkage), our model also needs to be validated in another independent larger cohort of patients surviving AHRF.

In conclusion, AHRF survivors have distinct predictors for OSA. Our data suggest that systematic pulmonary function testing is useful in this population to assess important conditions such as COPD and to raise the suspicion of comorbid OSA.

## Supporting information

S1 Dataset (XLSX)

## References

[1] Vestbo J, Hurd SS, Agusti AG, Jones PW, Vogelmeier C, Anzueto A, et al Global strategy for the diagnosis, management, and prevention of chronic obstructive pulmonary disease: GOLD executive summary. Am J Respir Crit Care Med. 2013;187(4):347–65. Epub 2012/08/11. 10.1164/rccm.201204-0596PP . 22878278

[2] Malhotra A, Orr JE, Owens RL. On the cutting edge of obstructive sleep apnoea: where next? The Lancet Respiratory Medicine. 2015;3(5):397–403. 10.1016/S2213-2600(15)00051-X. 25887980 PMC4431916

[3] Levy P, Kohler M, McNicholas WT, Barbe F, McEvoy RD, Somers VK, et al Obstructive sleep apnoea syndrome. Nat Rev Dis Primers. 2015;1:15015 Epub 2015/01/01. 10.1038/nrdp.2015.15 . 27188535

[4] Rochwerg B, Brochard L, Elliott MW, Hess D, Hill NS, Nava S, et al Official ERS/ATS clinical practice guidelines: noninvasive ventilation for acute respiratory failure. European Respiratory Journal. 2017;50(2). 10.1183/13993003.02426-2016 28860265

[5] Davidson AC, Banham S, Elliott M, Kennedy D, Gelder C, Glossop A, et al BTS/ICS guideline for the ventilatory management of acute hypercapnic respiratory failure in adults. Thorax. 2016;71(Suppl 2):ii1–ii35. 10.1136/thoraxjnl-2015-208209 26976648

[6] Carrillo A, Ferrer M, Gonzalez-Diaz G, Lopez-Martinez A, Llamas N, Alcazar M, et al Noninvasive ventilation in acute hypercapnic respiratory failure caused by obesity hypoventilation syndrome and chronic obstructive pulmonary disease. Am J Respir Crit Care Med. 2012;186(12):1279–85. Epub 2012/10/30. 10.1164/rccm.201206-1101OC . 23103736

[7] Adler D, Pepin JL, Dupuis-Lozeron E, Espa-Cervena K, Merlet-Violet R, Muller H, et al Comorbidities and Subgroups of Patients Surviving Severe Acute Hypercapnic Respiratory Failure in the Intensive Care Unit. Am J Respir Crit Care Med. 2017;196(2):200–7. Epub 2016/12/16. 10.1164/rccm.201608-1666OC . 27973930

[8] Piper AJ, Wang D, Yee BJ, Barnes DJ, Grunstein RR. Randomised trial of CPAP vs bilevel support in the treatment of obesity hypoventilation syndrome without severe nocturnal desaturation. Thorax. 2008;63(5):395–401. Epub 2008/01/22. 10.1136/thx.2007.081315 . 18203817

[9] Howard ME, Piper AJ, Stevens B, Holland AE, Yee BJ, Dabscheck E, et al A randomised controlled trial of CPAP versus non-invasive ventilation for initial treatment of obesity hypoventilation syndrome. Thorax. 2017;72(5):437–44. Epub 2016/11/18. 10.1136/thoraxjnl-2016-208559 . 27852952

[10] Köhnlein T, Windisch W, Köhler D, Drabik A, Geiseler J, Hartl S, et al Non-invasive positive pressure ventilation for the treatment of severe stable chronic obstructive pulmonary disease: a prospective, multicentre, randomised, controlled clinical trial. The Lancet Respiratory Medicine. 2014;2(9):698–705. 10.1016/S2213-2600(14)70153-5. 25066329

[11] Murphy PB, Rehal S, Arbane G, et al Effect of home noninvasive ventilation with oxygen therapy vs oxygen therapy alone on hospital readmission or death after an acute copd exacerbation: A randomized clinical trial. JAMA. 2017;317(21):2177–86. 10.1001/jama.2017.4451 28528348 PMC5710342

[12] Machado M-CL, Vollmer WM, Togeiro SM, Bilderback AL, Oliveira M-VC, Leitão FS, et al CPAP and survival in moderate-to-severe obstructive sleep apnoea syndrome and hypoxaemic COPD. European Respiratory Journal. 2010;35(1):132–7. 10.1183/09031936.00192008 19574323

[13] Marin JM, Soriano JB, Carrizo SJ, Boldova A, Celli BR. Outcomes in patients with chronic obstructive pulmonary disease and obstructive sleep apnea: the overlap syndrome. Am J Respir Crit Care Med. 2010;182(3):325–31. Epub 2010/04/10. 10.1164/rccm.200912-1869OC . 20378728

[14] McNicholas WT. COPD-OSA Overlap Syndrome. Chest. 2017. 10.1016/j.chest.2017.04.160. 28442310

[15] McNicholas WT. Chronic Obstructive Pulmonary Disease and Obstructive Sleep Apnea. American Journal of Respiratory and Critical Care Medicine. 2009;180(8):692–700. 10.1164/rccm.200903-0347PP . 19628778

[16] Weitzenblum E, Chaouat A, Kessler R, Canuet M. Overlap syndrome: obstructive sleep apnea in patients with chronic obstructive pulmonary disease. Proc Am Thorac Soc. 2008;5(2):237–41. Epub 2008/02/06. 10.1513/pats.200706-077MG . 18250217

[17] Chaouat A, Weitzenblum E, Krieger J, Ifoundza T, Oswald M, Kessler R. Association of chronic obstructive pulmonary disease and sleep apnea syndrome. Am J Respir Crit Care Med. 1995;151(1):82–6. Epub 1995/01/01. 10.1164/ajrccm.151.1.7812577 . 7812577

[18] Flenley DC. Sleep in chronic obstructive lung disease. Clin Chest Med. 1985;6(4):651–61. Epub 1985/12/01. . 2935359

[19] Shawon MS, Perret JL, Senaratna CV, Lodge C, Hamilton GS, Dharmage SC. Current evidence on prevalence and clinical outcomes of co-morbid obstructive sleep apnea and chronic obstructive pulmonary disease: A systematic review. Sleep Med Rev. 2017;32:58–68. Epub 2017/02/09. 10.1016/j.smrv.2016.02.007 . 28169105

[20] Berry RB, Budhiraja R, Gottlieb DJ, Gozal D, Iber C, Kapur VK, et al Rules for scoring respiratory events in sleep: update of the 2007 AASM Manual for the Scoring of Sleep and Associated Events. Deliberations of the Sleep Apnea Definitions Task Force of the American Academy of Sleep Medicine. J Clin Sleep Med. 2012;8(5):597–619. Epub 2012/10/16. 10.5664/jcsm.2172 23066376 PMC3459210

[21] Heinzer R, Vat S, Marques-Vidal P, Marti-Soler H, Andries D, Tobback N, et al Prevalence of sleep-disordered breathing in the general population: the HypnoLaus study. The Lancet Respiratory Medicine. 2015;3(4):310–8. 10.1016/S2213-2600(15)00043-0. 25682233 PMC4404207

[22] Marti-Soler H, Hirotsu C, Marques-Vidal P, Vollenweider P, Waeber G, Preisig M, et al The NoSAS score for screening of sleep-disordered breathing: a derivation and validation study. The Lancet Respiratory Medicine. 4(9):742–8. 10.1016/S2213-2600(16)30075-3 27321086

[23] Pellegrino R, Viegi G, Brusasco V, Crapo RO, Burgos F, Casaburi R, et al Interpretative strategies for lung function tests. Eur Respir J. 2005;26(5):948–68. Epub 2005/11/03. 10.1183/09031936.05.00035205 . 16264058

[24] Chung F, Subramanyam R, Liao P, Sasaki E, Shapiro C, Sun Y. High STOP-Bang score indicates a high probability of obstructive sleep apnoea. BJA: British Journal of Anaesthesia. 2012;108(5):768–75. 10.1093/bja/aes022 22401881 PMC3325050

[25] Hoerl AE, Kennard RW. Ridge regression: Biased estimation for nonorthogonal problems. Technometrics. 1970;12(1):55–67.

[26] Hurvich CM, Tsai C-L. Regression and time series model selection in small samples. Biometrika. 1989;76(2):297–307.

[27] Ambler G, Brady AR, Royston P. Simplifying a prognostic model: a simulation study based on clinical data. Statistics in medicine. 2002;21(24):3803–22. 10.1002/sim.1422 12483768

[28] Moons K, Donders ART, Steyerberg E, Harrell F. Penalized maximum likelihood estimation to directly adjust diagnostic and prognostic prediction models for overoptimism: a clinical example. Journal of clinical epidemiology. 2004;57(12):1262–70. 10.1016/j.jclinepi.2004.01.020 15617952

[29] Harrell FE. rms: Regression Modeling Strategies. 2017.

[30] Dempsey JA, Veasey SC, Morgan BJ, O’Donnell CP. Pathophysiology of Sleep Apnea. Physiological Reviews. 2010;90(1):47–112. 10.1152/physrev.00043.2008 20086074 PMC3970937

[31] Heinzer RC, Stanchina ML, Malhotra A, Jordan AS, Patel SR, Lo YL, et al Effect of increased lung volume on sleep disordered breathing in patients with sleep apnoea. Thorax. 2006;61(5):435–9. Epub 2006/02/24. 10.1136/thx.2005.052084 . 16490766 PMC2111199

[32] Heinzer RC, Stanchina ML, Malhotra A, Fogel RB, Patel SR, Jordan AS, et al Lung volume and continuous positive airway pressure requirements in obstructive sleep apnea. Am J Respir Crit Care Med. 2005;172(1):114–7. Epub 2005/04/09. 10.1164/rccm.200404-552OC . 15817803 PMC2718445

[33] Squier SB, Patil SP, Schneider H, Kirkness JP, Smith PL, Schwartz AR. Effect of end-expiratory lung volume on upper airway collapsibility in sleeping men and women. Journal of Applied Physiology. 2010;109(4):977–85. 10.1152/japplphysiol.00080.2010 20576839 PMC2963333

[34] Tagaito Y, Isono S, Remmers JE, Tanaka A, Nishino T. Lung volume and collapsibility of the passive pharynx in patients with sleep-disordered breathing. Journal of Applied Physiology. 2007;103(4):1379–85. 10.1152/japplphysiol.00026.2007 17600160

[35] Biselli PJC, Grossman PR, Kirkness JP, Patil SP, Smith PL, Schwartz AR, et al The Effect of Increased Lung Volume in Chronic Obstructive Pulmonary Disease on Upper Airway Obstruction during Sleep. Journal of Applied Physiology. 2015 10.1152/japplphysiol.00455.2014 26048975 PMC4526705

[36] Krachman SL, Tiwari R, Vega ME, Yu D, Soler X, Jaffe F, et al Effect of Emphysema Severity on the Apnea–Hypopnea Index in Smokers with Obstructive Sleep Apnea. Annals of the American Thoracic Society. 2016;13(7):1129–35. 10.1513/AnnalsATS.201511-765OC 27078132 PMC5015748

[37] Soler X, Liao SY, Marin JM, Lorenzi-Filho G, Jen R, DeYoung P, et al Age, gender, neck circumference, and Epworth sleepiness scale do not predict obstructive sleep apnea (OSA) in moderate to severe chronic obstructive pulmonary disease (COPD): The challenge to predict OSA in advanced COPD. PLoS One. 2017;12(5):e0177289 Epub 2017/05/17. 10.1371/journal.pone.0177289 . 28510598 PMC5433709

[38] Bailly S, Destors M, Grillet Y, Richard P, Stach B, Vivodtzev I, et al Obstructive Sleep Apnea: A Cluster Analysis at Time of Diagnosis. PLoS One. 2016;11(6):e0157318 Epub 2016/06/18. . 27314230 10.1371/journal.pone.0157318 PMC4912165

[39] Gagnadoux F, Le Vaillant M, Paris A, Pigeanne T, Leclair-Visonneau L, Bizieux-Thaminy A, et al Relationship Between OSA Clinical Phenotypes and CPAP Treatment Outcomes. CHEST. 149(1):288–90. 10.1016/j.chest.2015.09.032 26757296

[40] Ye L, Pien GW, Ratcliffe SJ, Björnsdottir E, Arnardottir ES, Pack AI, et al The different clinical faces of obstructive sleep apnoea: a cluster analysis. European Respiratory Journal. 2014;44(6):1600–7. 10.1183/09031936.00032314 25186268 PMC6675398

[41] Corral-Peñafiel J, Pepin J-L, Barbe F. Ambulatory monitoring in the diagnosis and management of obstructive sleep apnoea syndrome. European Respiratory Review. 2013;22(129):312–24. 10.1183/09059180.00004213 23997059 PMC9487363

[42] Soler X, Gaio E, Powell FL, Ramsdell JW, Loredo JS, Malhotra A, et al High Prevalence of Obstructive Sleep Apnea in Patients with Moderate to Severe Chronic Obstructive Pulmonary Disease. Ann Am Thorac Soc. 2015;12(8):1219–25. Epub 2015/04/15. 10.1513/AnnalsATS.201407-336OC . 25871443 PMC5466175

